# Improving Pediatric Hypertension Screening in an Academic Primary Care Setting

**DOI:** 10.1097/pq9.0000000000000746

**Published:** 2024-07-10

**Authors:** Vildan Tas, Esma Birisci, Rachel Achor Jones, John J. Forbus, Richard T. Blaszak, Brendan Crawford, Mohammad Ilyas, James S. Magee, Laura L. Sisterhen

**Affiliations:** From the * Department of Pediatrics, University of Pittsburg Medical Center Children’s Hospital of Pittsburgh, Little Rock, Penn.; † Department of Econometrics, Bursa Uludağ University, Bursa, Turkey; ‡ Process Improvement and Population Health Departments, Arkansas Children’s Hospital, Little Rock, Ark.; § Department of Pediatrics, University of Arkansas for Medical Sciences, Little Rock, Ark.

## Abstract

**Introduction:**

Adherence to the American Academy of Pediatrics clinical practice guidelines for screening and managing high blood pressure (BP) is low. This team sought to improve recognition and documentation of relevant diagnoses in patients aged 13–20 years who presented to general pediatric clinics.

**Methods:**

The primary outcome measure was the proportion of office visits for patients ages 13–20 with a BP ≥ 120/80 with a visit or problem list diagnosis of hypertension or elevated BP. Secondary measures included (1) the proportion of patients who had their BP measured in the right arm, (2) the proportion of patients who had a mid-arm circumference measurement recorded, and (3) the proportion of patients who had a second BP reading measured at the visit. Interventions addressed key drivers for evidence-based high BP screening: standard BP measurement, electronic health record clinical decision support, and clinical pathway adoption. Data were collected over a twenty-seven-month period and plotted using the Laney p’ chart.

**Results:**

Provider documentation of elevated BP or hypertension improved from a baseline mean of 24% in April 2020 through January 2022 to 41% in February 2021 through June 2022. All secondary outcome measures also demonstrated significant improvement.

**Conclusions:**

This project demonstrates the feasibility of improving adherence to best practices of BP measurement in primary care clinics through education, acquisition of resources, and implementation of electronic health record flags for abnormal values.

## INTRODUCTION

Hypertension in childhood and adolescence is associated with an increased risk of cardiovascular disease. Left ventricular hypertrophy, a risk factor for adult-onset cardiovascular disease, is already present in 30% of children with hypertension.^1^ The global estimate of childhood hypertension is 4%, and rising rates of obesity suggest a risk for increased incidence of hypertension.^2,3^ In 2017, the American Academy of Pediatrics updated its clinical practice guideline for the management of hypertension in children and adolescents, emphasizing the importance of the primary care provider in diagnosing and managing hypertension.^4^ However, hypertension is often under or misdiagnosed in primary care settings.^5,6^

The American Academy of Pediatrics clinical practice guideline outlines best practices to improve accuracy and consistency in blood pressure (BP) measurement. First, the right arm should be selected to allow comparison with normative standards (unless abnormal cardiac anatomy). Second, the mid-arm circumference should be measured to select an appropriately sized cuff. Last, an isolated elevated oscillatory BP measurement should be followed by repeated measurements during the same visit and averaged to determine the patient’s BP category. The category or BP level determines the future BP screening schedule. A BP of ≥120/80 mm Hg is considered abnormal in children aged 13 or older.^4^

### Review of Current Evidence

There is limited literature on implementing the American Academy of Pediatrics hypertension guideline in primary care settings. Single and multi-site quality improvement (QI) projects published in the last 2 years demonstrated low adherence to the guideline.^5,7^ In the multi-site study, among the 2677 patient’s charts reviewed, only 2% had all the BP measurement steps completed, and 10% had an appropriate diagnosis documented in the medical record.^5^

### Local Problem

Our institution’s baseline data assessing adherence to best practices for measuring BP were low. Measurement of mid-arm circumference was not a customary practice. The electronic health record did not have a standardized location to document the mid-arm circumference measurement. Also, few patients had repeated BP measurements during each clinic encounter. Finally, provider documentation of an elevated BP or hypertension in the electronic health record (visit or problem list diagnosis) was low. Therefore, the team sought to improve the recognition and documentation of high BP as a visit diagnosis in patients aged 13–20 years who presented to the general pediatric clinics.

### Setting

The improvement project was conducted at three general pediatric clinics at an urban, academic, free-standing children’s hospital, from April 2020 through June 2022. The nonteaching service clinic is staffed by advanced practice registered nurses and pediatrician faculty from the Department of Pediatrics. The other two continuity and teaching clinics are staffed with medical students and residents supervised by faculty. Approximately 20 faculty physicians staff the three clinic sites. The patient race/ethnicity distributions are approximately 49% Black, 25% White, and 25% Hispanic. The majority have Medicaid as the primary payer. Epic is our electronic health record.

Our institutional standard practice is to obtain a BP measurement uniformly at all well-child visits for ages three and over and all ADHD encounters. BP measurements must be requested by a provider for any other visit type.

Our institutional review board reviewed the project and determined it was not human subject research as defined in 45 CFR 46.102. Therefore, it did not fall under the authority of the IRB (institutional review board) review process.

## METHODS

**Fig. 1.**
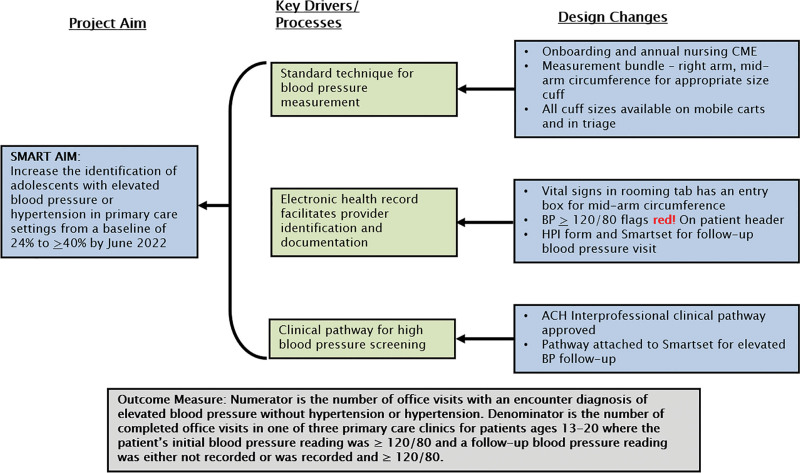
Key driver diagram. CME, continuing medical education; SMART, Specific, Measurable, Achievable, Realistic, and Timely.

Input from stakeholders, including providers, nurses, and clinical operations managers, was periodically obtained and used to design interventions. Analysis of the current state revealed the primary drivers of evidence-based high BP screening were nurse adherence to a BP measurement bundle, clinical decision support, and interprofessional pathway adoption (Fig. 1). We conducted monthly improvement team meetings with pediatric nephrologists, primary care provider champions, and clinical nurse educators to review the plan-do-study-act (PDSA) cycles. Each of the three clinics identified a provider champion responsible for the local site’s workflow and problem-solving in real time.

## PDSA CYCLES (IMPLEMENTATION STRATEGIES)

The primary outcome measure was the percentage of office visits for patients aged 13–20 with a BP ≥ 120 mm Hg systolic or ≥80 mm Hg diastolic who had an associated visit or problem list diagnosis of hypertension or elevated BP (including ICD-10-related diagnoses).^8^ Patients were excluded from the denominator if a second measurement within the visit was normal.

Secondary (process) measures included (1) the percentage of office visits for patients aged 13–20 with an initial BP reading ≥120/80 where the BP was measured in the right arm, (2) the percentage of office visits for patients ages 13–20 with an initial BP reading ≥120/80 where a mid-arm circumference measurement was recorded at the visit and (3) the percentage of office visits for patients aged 13–20 with an initial BP reading ≥120/80, where a second BP reading (auscultatory) was measured at the visit. The scope of the QI project was limited to obtaining at least one repeat auscultatory BP, as there was no buy-in for more than two in one visit due to perceived time restraint. A BP measurement “bundle” criterion was met if all three components were present: right arm, mid-arm circumference, and repeat BP.

The project aimed to increase provider documentation of elevated BP or hypertension in patients aged 13-–20 with a BP of ≥120/80 from a baseline mean of 24% to ≥40% by June 2022. Interventions were designed to address three key drivers for evidence-based high BP screening: nursing education on the BP measurement bundle, electronic health record clinical decision support, and cross-functional, interprofessional clinical pathway adoption (Fig. 1). Interventions conformed to standard PDSA cycles in the Model for Improvement framework.^9^

The annual training of clinical nursing staff on evidence-based BP measurement techniques began in November 2020 (PDSA Cycle 1). The annual Clinical Skills Fair is required for all primary care nursing staff. Training includes a presentation on the importance of hypertension screening and a demonstration of accurate BP measurement. Each nurse is observed and “checked off” on their clinical skills by a clinical nurse educator. Newly hired nursing staff receive BP measurement education from preceptors between clinical skills events. A clinical nurse educator began conducting rounds and quality huddles with nurses in clinic in January 2022. Nurses identified resources and equipment to facilitate practice changes. These included new chairs with arm rests, tape measures for mid-arm circumference measurement on every BP machine, and standardized BP cuff brands and models in each triage room. Previously, the clinic stocked several brands and models of BP cuffs, each of which had different connectors, making it difficult for nurses to find matched equipment. The BP cuffs were placed to the right of the patient’s chair in triage as a visual cue when possible. Many sizes of BP cuffs, including thigh cuffs, were made available in each clinic triage area to fit the range of mid-arm circumference measurements.

Faculty education on best practices in identification and diagnosis of hypertension occurred in March 2021, and providers received continuing medical education credit for attending the one-hour lecture (PDSA Cycle 2). A general pediatrician and pediatric nephrologist led the presentation for the institution. Residents received annual education in the continuity clinic that began in October 2021.

**Fig. 2.**
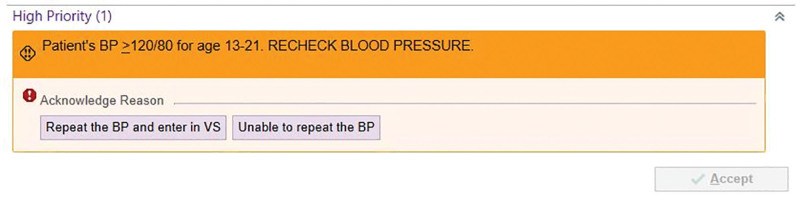
Best Practice Advisory, epic electronic health record. VS, vital signs.

In collaboration with the Associate Medical Information Officer and data analysts, the team implemented a best practice advisory (Fig. 2) to alert staff of an elevated BP reading during the rooming process (PDSA Cycle 3). The best practice advisory is active for triaging staff working in the primary care division. A pop-up message with an orange banner displays “Patient’s BP ≥120/80 for ages 13–21. Recheck BP.” An “Acknowledge Reason” is displayed below with two options to select: “Repeat the BP and enter in Vital Signs” or “Unable to repeat the BP.” A comment section appears after selecting a button. A comment is only required if unable to obtain a second reading. The team also reviewed the thresholds set for the BP to display in red on the patient’s header. From the patient’s header, users can quickly review a patient’s vital signs in a compact space available throughout the chart without leaving their workflows. In June 2021, the BP thresholds that flag measurements with a red exclamation mark were adjusted for consistency with national guideline’s recommended screening thresholds (PDSA Cycle 4).^4^ During the project, the team discovered that the maximum systolic warning was set at 125 mm Hg for ages 13 and over, and there were no recommended cutoffs for diastolic BP. These settings had been changed from the electronic health record’s foundation settings, which have an age override list. During the project, the team requested adjustment to the maximum systolic and diastolic warnings to 120 mm Hg and 80 mm Hg, respectively, for ages ≥ 13. This change required amendment to the Vital Signs measurement hospital policy, and nursing education throughout the hospital.

The division of general pediatrics, in collaboration with pediatric nephrology, developed a local pathway for high BP screening during health supervision exams. The pathway begins with obtaining BP at all well visits for ages three and over during triage. If the initial oscillatory BP is high, the nurse will receive an immediate message in the electronic health record to repeat an auscultatory BP in the right arm with an appropriate size cuff after 3–5 minutes of the child sitting quietly. Triaging staff document the mid-arm circumference during the rooming process in the vital signs section of the electronic health record. The pathway was approved by the hospital’s Patient Care Oversight Committee in January 2022 and made available within the electronic health record (PDSA Cycle 5).

## STATISTICAL ANALYSES

Data were plotted on Laney p charts using Microsoft Excel for Microsoft 365 MSO (Version 2308 Build 16.0.16731.20310). Centerline shifts were created when there were eight consecutive points above the centerline. The Laney p chart is used for large data sets with high variation within measures and focuses on the variation between data.^10^

## RESULTS

**Fig. 3.**
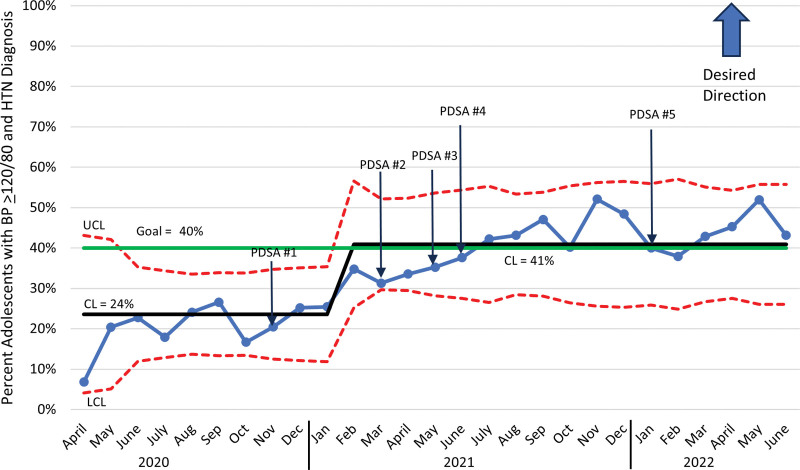
Monthly statistical process control chart showing percentage of visits with a diagnosis of elevated BP or hypertension (HTN) in patients aged 13–20 whose BP reading was ≥120/80. CL, centerline; LCL, lower control limit; UCL, upper control limit.

There were 5926 primary care office visits for patients aged 13–20 where the initial BP was ≥ 120/80 in the 27-month period between April 1, 2020, and June 30, 2022. Multiple iterative PDSA cycles significantly improved adherence to the BP measurement bundle and provider documentation of elevated BP and hypertension. Provider documentation of elevated BP or hypertension in the visit diagnoses improved from a baseline mean of 24% April 2020 through January 2021 to 41% in February 2021 through June 2022 (Fig. 3).

**Fig. 4.**
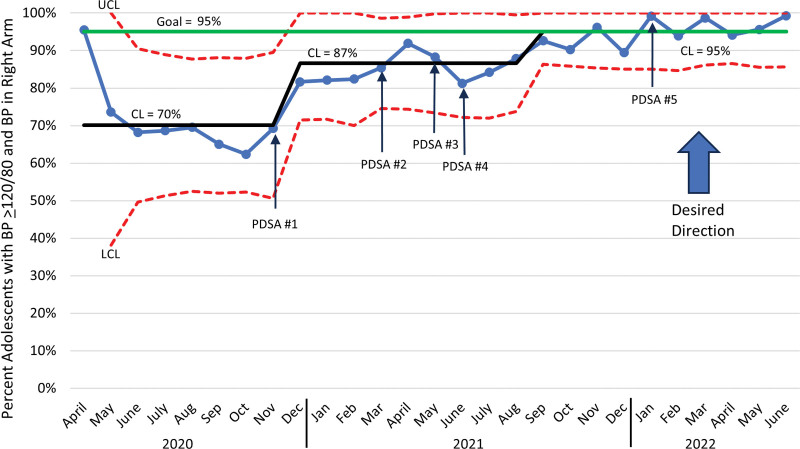
Monthly statistical process control chart showing percentage of visits with BP measured in right arm in patients ages 13–20 whose BP reading was ≥120/80.

**Fig. 5.**
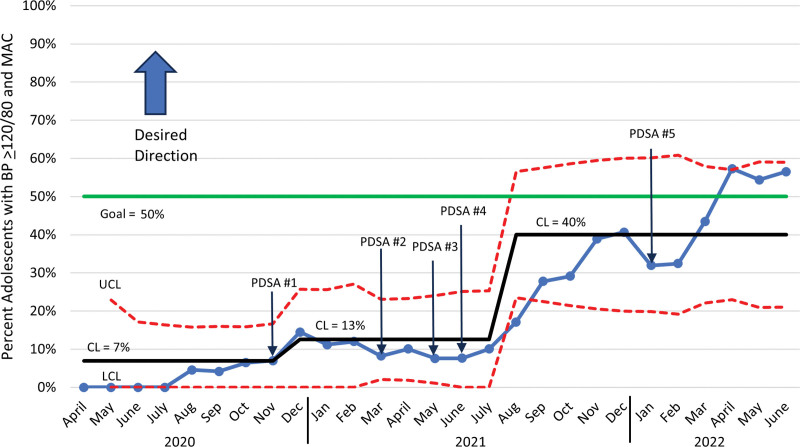
Monthly statistical process control chart showing percentage of visits with mid-arm circumference documented in patients ages 13–20 whose BP reading was ≥120/80.

**Fig. 6.**
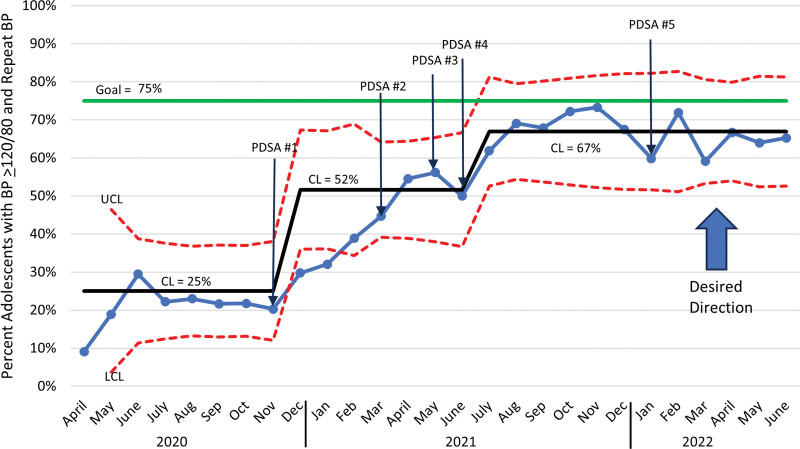
Monthly statistical process control chart showing percentage of visits with a BP repeated using auscultatory technique in patients ages 13–20 whose BP reading was ≥120/80.

Nurses significantly improved adherence to the measurement bundle. The percentage of patients with abnormal BP who had their BP measured in the right arm improved from a baseline mean of 70% with a centerline shift in December 2020 to 87% and another centerline shift in September 2021 to 95% (Fig. 4). Documentation of mid-arm circumference measurement improved from a baseline mean of 7% with a centerline shift to 13% in December 2020 and another centerline shift to 40% in August 2021 (Fig. 5). The percentage of adolescents with an elevated BP who had a second BP measurement improved from a baseline mean of 25% with a centerline shift to 52% in December 2020 and another shift to 67% in July 2021 (Fig. 6). Adherence to the bundle, or all three components, improved from a baseline of 3% with a centerline shift to 8% in November 2020 and another centerline shift to 32% in July 2021 (**See figure, Supplemental Digital Content 1,** which shows the percentage bundle compliance for adolescents with BP ≥ 120/80. http://links.lww.com/PQ9/A569).

## DISCUSSION

This project demonstrated modest improvement in problem-based documentation of elevated BP above baseline. However, there is still an opportunity for improvement in the providers’ clinical documentation of elevated BP or hypertension. This initiative also demonstrates the feasibility of improving mid-arm circumference measurement and repeating the BP if elevated. Potential barriers to diagnosis include lack of familiarity with the guidelines, providers not agreeing with the guideline recommendations, confusion with conflicting recommendations from the US Preventative Services Taskforce and the AAP, and limited access to ambulatory BP monitoring and/or pediatric subspecialists.^5,11–13^

The findings of this QI project support the key action statement in the American Academy of Pediatrics’ clinical practice guideline that “organizations with EHRs used in an office setting should consider including flags for abnormal BP values, both when values are being entered and when they are being viewed.” The EHR interventions included the best practice advisory, and the adjustment of thresholds for a value to flag red when BPs entered were ≥120/80 mm Hg for patients aged 13 and older. The annotated control chart (Fig. 3) shows that these two interventions were associated with the greatest improvement in our metrics. Our institution uses the Epic electronic health record, whereas another institution demonstrated similar successes with integration into Centricity. However, that QI project included a small sample size of 30–40 random charts each improvement cycle and required individualized feedback, which may be difficult to sustain.^7^ Meisner et al conducted a retrospective review for encounter diagnoses of hypertension in health maintenance exams in patients ages 3–17 after the implementation of a similar clinical decision support tool within Epic. Diagnosis rates improved from 17.9% to 33.7% with the clinical decision support tool. Provider recognition improved to 75.5% if the systolic BP remained ≥95^th^ percentile upon repeat measurement. The authors concluded that interventions focused on repeat BP at medical intake would have greater impact on provider recognition of hypertension.^14^ Our study builds upon this by assessing the mid-arm circumference measurement at the time of medical intake with repeat BP measurement, in three different clinics. In contrast, our study included unwell visits and evaluated provider recognition of elevated BP as well as hypertension classification. This may explain the lower rate of improvement in visit diagnosis.

The improvement team utilized several implementation strategies for evidence-based practice.^15^ By highlighting the problem of pediatric hypertension and the impact of high-quality BP measurement during annual continuing education, as well as staff meetings, unit newsletters, and unit huddles, the team created awareness and interest among nurses. To build knowledge and commitment, the team identified change agents in each clinic, conducted continuing medical education, performed gap assessments, and enlisted clinician input to create a clinical pathway. Ongoing education in rotation was necessary for new learners in the teaching clinics. The team promoted evidence-based practices through electronic health record flags such as the best practice advisory and nonpunitive discussion of performance results. Finally, the team pursued integration and sustained use by celebrating clinic progress toward goals, auditing, and giving feedback to the clinic medical directors.

Barriers that the team addressed included nursing cross-coverage. Nursing shortages during the coronavirus disease 2019 pandemic required more nonprimary care team members to work in primary care clinics. They were less familiar and efficient with the rooming process and the expected BP measurement bundle. In discussion with stakeholders, the team did not pursue the determination of three BP measurements in one primary care visit encounter as there was no team buy-in for this practice. The modified measurement algorithm, an additional consensus opinion recommendation in the national guidelines,^4^ is perceived as too demanding for clinical staff in primary care without the tools to calculate an average between two measurements quickly. Following the algorithm could result in five measurements, three oscillometric and two auscultatory, with the last two auscultatory measurements averaged to classify a patient during one encounter. Limited time for well visits with multiple screening recommendations for vision, hearing, developmental, and mental health is a perceived barrier. The locally adopted pathway required one repeat BP using auscultatory technique. Providers determined interventions and follow-ups based on the classification of abnormal BP. Interventions included physical activity and nutrition counseling. Ambulatory BP monitoring was recommended if the BP was elevated for three visits. If the ambulatory BP monitoring was abnormal, a diagnostic evaluation was outlined in the pathway. This practice can potentially increase healthcare utilization through subspecialty referral or ambulatory BP monitoring. However, many patients with elevated BP also had other diagnoses that required frequent follow-up, such as ADHD or overweight/obesity. Future QI work will be necessary to expand the scope to include accurate BP categorization and the determination of a screening schedule according to the guidelines. Further, BP was not obtained in all visit encounters. Future work should create processes where a BP is routinely obtained for indications other than well visits and ADHD, such as all visits with patients who have obesity or a history of diabetes, chronic kidney disease, congenital heart disease, or prematurity. Finally, QI work on population health management, including follow-up, treatment, and control of hypertension, for children identified with hypertension is needed.

In conclusion, measuring BP at routine well-child visits enables the early detection of primary hypertension as well as the detection of asymptomatic hypertension secondary to another underlying disorder. QI projects utilizing PDSA cycles, including a diagnostic pathway to address barriers in pediatric primary care, are important in identifying those children who may benefit from early recognition and treatment of high BP.

**Fig. 7.**
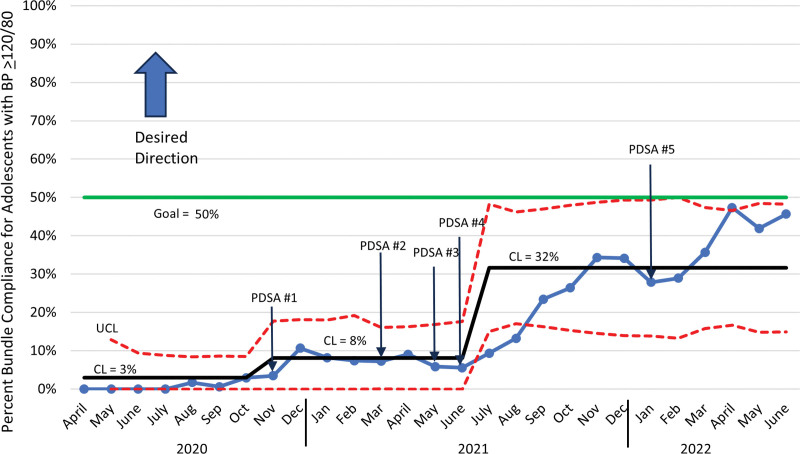
Monthly statistical process control chart showing percentage of visits with BP bundle adherence (right arm, mid-arm circumference, repeat BP) in patients ages 13–20 whose BP reading was ≥120/80.

## ACKNOWLEDGMENTS

The authors thank the following persons for assistance with the study: Debra Becton, MD, Director of Continuity Clinic; Maria Eilers, MSN, RN, CPN, Clinical Nurse Educator; Linda Mars, RN and Kelley Means, BSN, RN, CPN, Clinical Operations managers; Annmarie Neal, BSN, RN, CPN; Marcus O’Brien, MD; and Alma Floyd, BSN, RN-BC, Epic Application Coordinator.

## Supplementary Material


