# Supplementary figures and images for: Improving Pediatric Hypertension Screening in an Academic Primary Care Setting
Source: Pediatr Qual Saf. 2024 Jul 10;9(4):e746. doi: 10.1097/pq9.0000000000000746 (PMC11236397; doi:10.1097/pq9.0000000000000746)

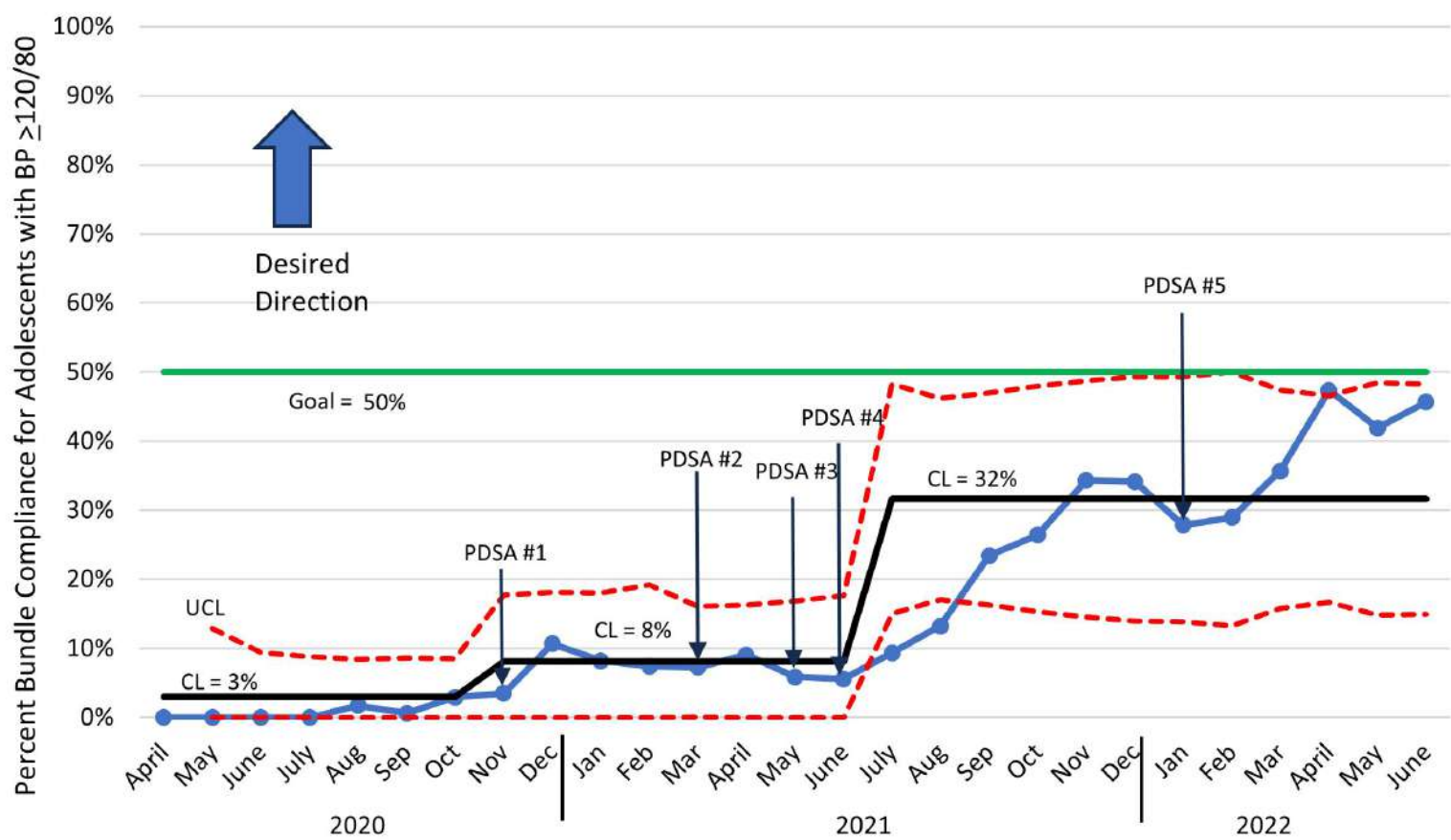

Supplement: Supplementary file 1 [file pqs-9-e746-s001.pdf]
